# Supplementary material for: Replacement of the Genomic Scaffold Improves the Replication Efficiency of Synthetic Klebsiella Phages
Source: Int J Mol Sci. 2025 Jul 16;26(14):6824. doi: 10.3390/ijms26146824 (PMC12295037; doi:10.3390/ijms26146824)
Supplement: Supplementary file 1 [file ijms-26-06824-s001.zip › ijms-3719236-supplementary.pdf]

Supplementary Materials

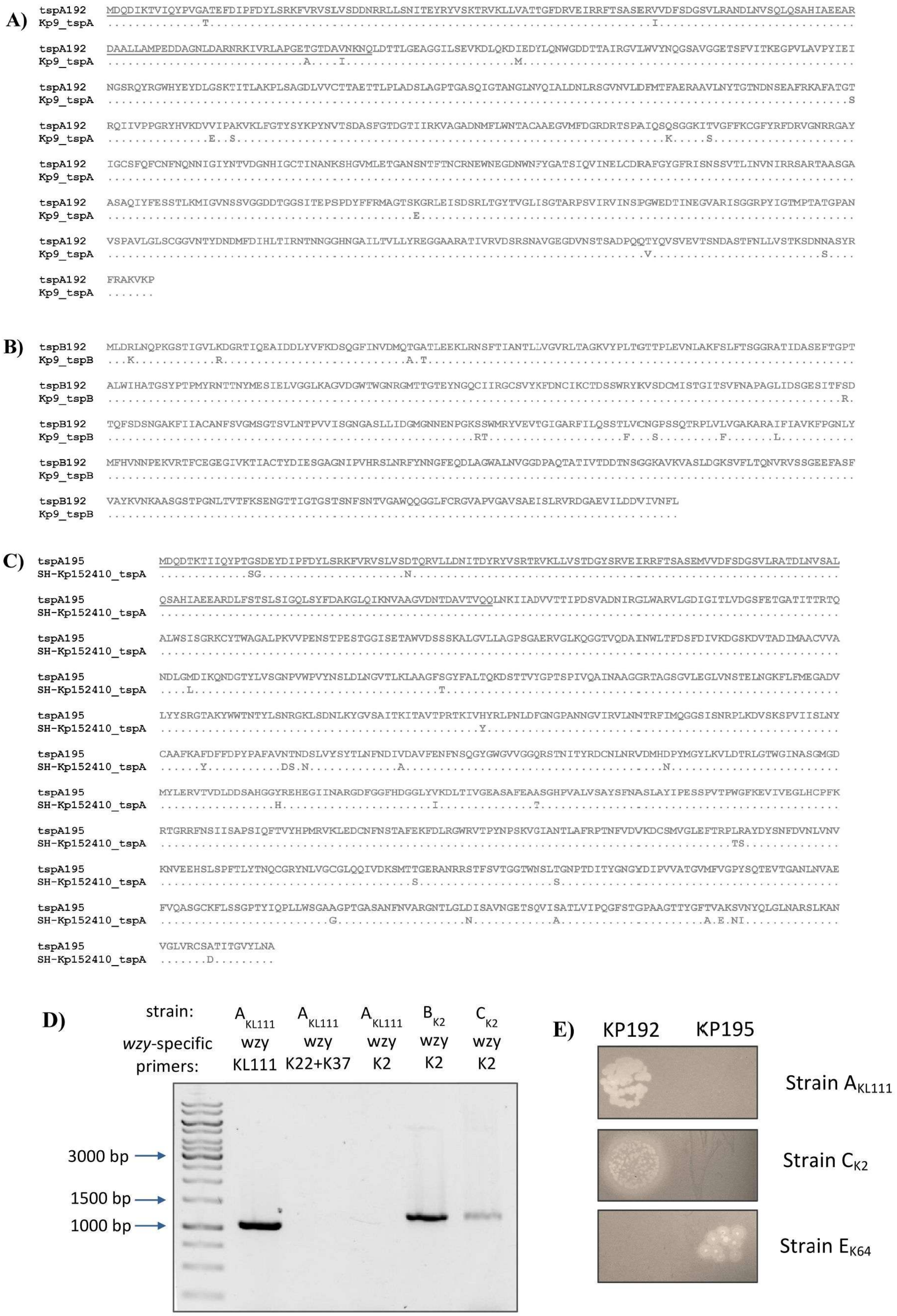

Figure S1. K-type specificity of KP192 and KP195 phages

(A) Amino acid alignment of proteins tspA192 from phage KP192 and gp42 from phage Kp9 showing 98% identity. The fragment corresponding to the T7gp17-like N-terminal anchoring domain is underlined. (B) Amino acid alignment of proteins tspB192 from phage KP192 and gp43 from phage Kp9 (the tailspike specific to K2 capsule type) showing 98% identity. (C) Amino acid alignment of proteins tspA195 from phage KP195 and gp42 from phage SH-Kp 152410 (the tailspike specific to K64 capsule type) showing 97% identity. The fragment corresponding to the T7gp17-like N-terminal anchoring domain is underlined. (D) Electrophoregram of PCR products demonstrating that A_{KL111} strain belongs to the KL111 capsular type, while strains B_{K2} and C_{K2} belong to the K2 capsular type. ThermoFisher Scientific SM0311 ladder was used. 1.5 % agarose gel was stained using ethidium bromide and scanned under UV light (inverted image). (E) Plaques formed by phages KP192 and KP195 on lawns formed by *Klebsiella* strains A_{KL111}, C_{K2} and E_{K64}.

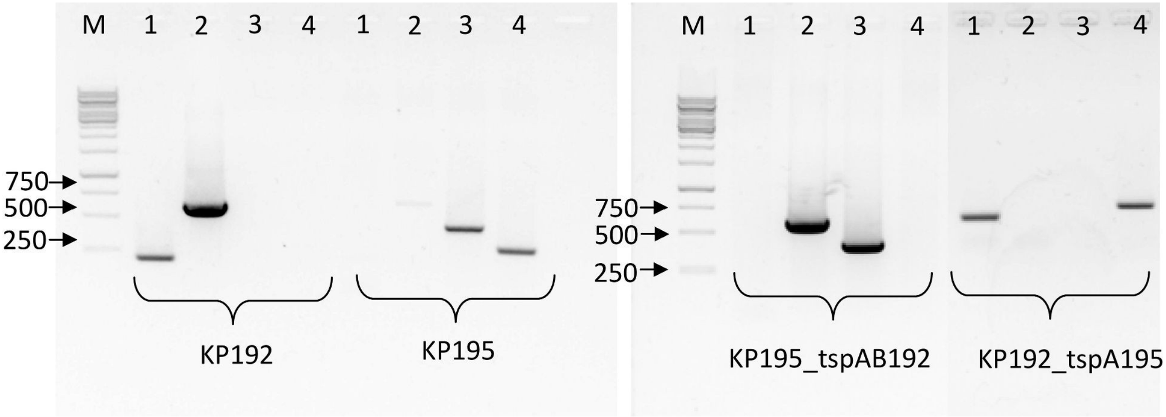

Figure S2. PCR validation of wild-type and synthetic phage genomes, related to the "Verification of genome assembly accuracy and genome sequencing" method

1 – lanes indicating the 206 bp PCR product obtained using KP192_10950bp_valid_dir and KP192_11110bp_valid_rev primers (see Table S1); 2 – lanes indicating the 621 bp PCR product obtained using KP192_tspB_36450bp_valid_dir and KP192_tspB_37050bp_valid_rev primers; 3 – lanes indicating the 411 bp PCR product obtained using KP195_9930bp_valid_dir and KP195_10320bp_valid_rev primers; 4 – lanes indicating the 270 bp PCR product obtained using KP195_tsp_36750_valid_dir and KP195_tsp_37000bp_valid_rev primers; M – DNA ladder GeneRuler 1kb (Thermo Fisher Scientific SM0311). 1.5 % agarose gel was stained using ethidium bromide and scanned under UV light (inverted image).

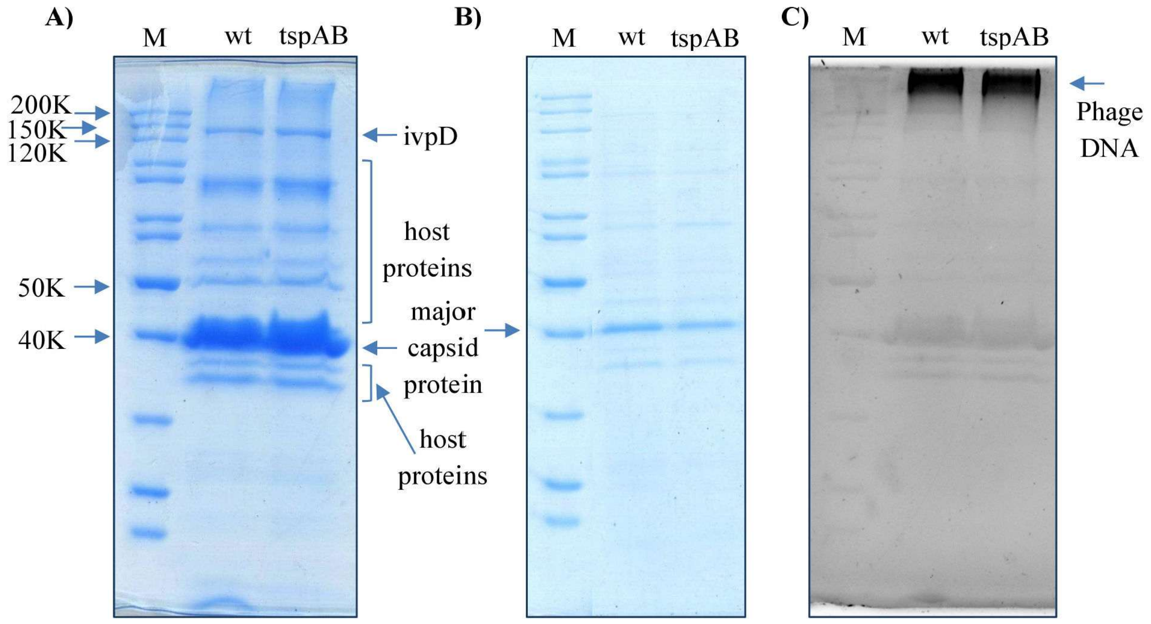

Figure S3. Determination of pseudo-physical titer of phage samples, related to "Determination of pseudo-physical titer (titer$_{PP}$) of phage samples" method

(A) Stained gel after denaturing protein electrophoresis (SDS-PAGE), 15 µl of phage samples applied (so that internal virion protein D (ivpD) band was visible); (B) same as in A, but 1.5 µl of samples applied (in order not to overload the major capsid protein band). (C) same gel as in A, additionally stained with ethidium bromide and scanned under UV light (inverted image) to detect phage genomic DNA. 12% (w/v) polyacrylamide gel containing SDS. M – protein ladder #26614 (Thermo Fisher Scientific), "wt" – KP192 phage, "tspAB" – KP195_tspAB192 phage.

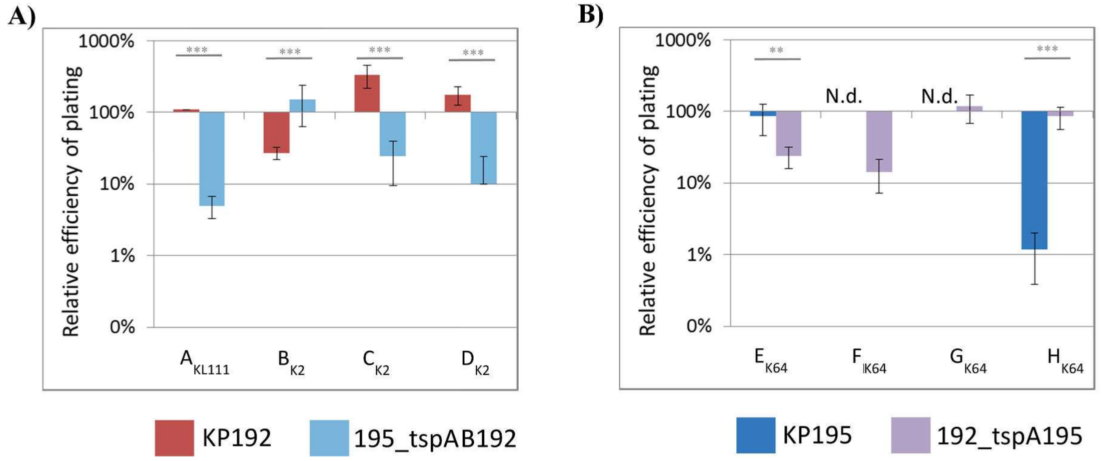

Figure S4. Relative efficiency of plating of synthetic phages

(A) Relative efficiency of plating of phages KP192 and 195_tspAB192 using *Klebsiella* strains A_{KL111}, B_{K2}, C_{K2} and D_{K2} (calculations were based on the data shown in Figure 3A). (B) Relative efficiency of plating of phages KP195 and 192_tspA195 using *Klebsiella* strains E_{K64}, F_{K64}, G_{K64} and H_{K64} (calculations were based on the data shown in Figure 3B). The efficiency of plaque formation by phage KP192 on strain A_{KL111} was chosen as a reference for all phage/strain combinations ($rEOP_{KP192@AKL111}$ = 100%). N.d. – not determined: phage plaques could not be counted due to their small size.

Table S1. Primers used for yeast colony screening, genome sequencing and phage sample validation, related to “Yeast colony screening” and “Verification of genome assembly accuracy and genome sequencing” methods and Figure S2.

**Primer name**	**Sequence**
KP192/5_40225_dir	5’TGGACTGGAGACTCTCGTGTGCTC3’
pRSII415_screening_rev	5’GATGAAAAGGACCCAGGTGGC3’
KP192_10950bp_valid_dir	5’TCTGTACAAATTATGGTCAGGCTAA3’
KP192_11110bp_valid_rev	5’ACCTACCTCGTGTAATACACCGATT3’
KP192_tspB_36450bp_valid_dir	5’CTTCTCAGACCAGACCGCTTGTGCT3’
KP192_tspB_37050bp_valid_rev	5’CACGAACACGGAGAGATATCTCA3’
KP195_9930bp_valid_dir	5’GTCGTCTGAATCCTGAAGCACT3’
KP195_10320bp_valid_rev	5’CTGCACCTCTCTTAATGTTG3’
KP195_tsp_36750_valid_dir	5’TGGTCCAACTGGAGCGAGTGCC3’
KP195_tsp_37000bp_valid_rev	5’TTCAGGTACACTCCGGTGATTGT3’
pt7_ivpD192/5_seq_dir	5’AAGGAGCTTGTTCCGAACGACCC3’
pt8_tspA192_seq_rev	5’TCATCTCCCCAGTTCTGTAGGTAATCC3’
pt8_tsp195_seq_v2_rev	5’GCTACCGTCAACAAGCGTGATTCCG3’

Table S2. Primers used for PCR-amplification of fragments of phage genomes, related to Figure 2B and "Preparation of PCR products for assembly of phage genomes" method.

Primer name	Sequence
pRSII415_192/5_genome_dir	5'GATACTATATGTTGATGTCTCTGTGTCCCTTGTCTCATGAGCGGATACATA3'
pRSII415_192/5_genome_rev	5'GGGGGATAACCAAAAGTGTAAACTGTGAGACCTTGTTCATGTGTGTTCAAAAAC3'
pt1_dir	5'TCTCACAGTTTACACTTTTGGT3'
pt1_rev	5'CATTCGAAGGTTCCCTATAG3'
pt2_dir	5'GCTGGGAATCTCTTTAAGGC3'
pt2_rev	5'CACCTACGTCCATGGTTGCCT3'
pt3_dir	5'GGTTAGTGTTCTCTTCGAGTCGC3'
pt3_rev	5'GCTGAGAAGTCCCAAGGTCG3'
pt4_dir	5'GCAGACATCGAGAAGCTGACC3'
pt4_rev	5'GCGCACAGGACTCTGCTCG3'
pt5_dir	5'GTCGTGGAGGACGTTGAGTC3'
pt5_rev	5'GGACCTGATACTCATAGCCGG3'
pt6_dir	5'GCCTGAAATCCTACGGTACCC3'
pt6_rev	5'AGTAGCTCCTTGTCGTAGCGG3'
pt7_dir	5'GACGTGGATGGTGATACCATTG3'
192_pt7_rev	5'ACCTCCTTTAGTTGGATGAGAAG3'
195_pt7_rev	5'GCCTCCTTAAGTTGAATGTGGAG3'
192_pt8_dir	5'CAGCCGAAGCCTAACTAATTAGG3'
195_pt8_dir	5'CAGCAGCCTAAACCTAACTAATT3'
pt8_rev	5'TCAGCCCCAGCGACCCCCG3'
pt9_dir	5'TATCCCTAGACTTCAACAACGAAG3'
pt9_rev	5'AGGGACACAGAGACATCAACATA3'

Table S3. Genomic DNA fragments used for phage genomes assembly and corresponding primers used for their amplification, related to Figure 2B and "Preparation of PCR products for assembly of phage genomes" method.

Fragment name	Primers used for amplification	Template DNA
Fragments of KP192 phage genome		
192_pt1	pt1_dir + pt1_rev	KP192 genomic DNA
192_pt2	pt2_dir + pt2_rev	
192_pt3	pt3_dir + pt3_rev	
192_pt4	pt4_dir + pt4_rev	
192_pt5	pt5_dir + pt5_rev	
192_pt6	pt6_dir + pt6_rev	
192_pt7	pt7_dir + 192_pt7_rev	
192_pt8	192_pt8_dir + pt8_rev	
192_pt9	pt9_dir + pt9_rev	
Fragments of KP195 phage genome		
195_pt1	pt1_dir + pt1_rev	KP195 genomic DNA
195_pt2	pt2_dir + pt2_rev	
195_pt3	pt3_dir + pt3_rev	
195_pt4	pt4_dir + pt4_rev	
195_pt5	pt5_dir + pt5_rev	
195_pt6	pt6_dir + pt6_rev	
195_pt7	pt7_dir + 195_pt7_rev	
195_pt8	195_pt8_dir + pt8_rev	
195_pt9	pt9_dir + pt9_rev	
vector fragment	pRSII415_192/5_genome_dir + pRSII415_192/5_genome_rev	pRSII-415 plasmid

Table S4. DNA fragment combinations used for assembly of phage genomes, related to Figure 2 and "Phage genome assembly in yeast" method.

Fragment name	KP192ctrl	KP192_tsp A195	KP195ctrl	KP195_tsp AB192
**Fragments of KP192 phage genome**				
192_pt1	+	+		
192_pt2	+	+		
192_pt3	+	+		
192_pt4	+	+		
192_pt5	+	+		
192_pt6	+	+		
192_pt7	+	+		
192_pt8	+			+
192_pt9	+	+		
**Fragments of KP195 phage genome**				
195_pt1			+	+
195_pt2			+	+
195_pt3			+	+
195_pt4			+	+
195_pt5			+	+
195_pt6			+	+
195_pt7			+	+
195_pt8		+	+	
195_pt9			+	+
**Vector fragment**	+	+	+	+

Table S5. Primers used for PCR-based capsule type determination of *Klebsiella* strains.

Primer name	Sequence
K2_wzy_typing_dir	5'GATTCGAAGAAAGTTTTCTCGAG3'
K2_wzy_typing_rev	5'AGTTGATGTCATTTTCGGCCCG3'
K22+37_wzy_typing_dir	5'CGGGAATTCCTGGAACAGCGCTG3'
K22+37_wzy_typing_rev	5'GCCTATGTACATTATTAATGCAAGGGC3'
KL111_wzy_typing_dir	5'GGTGCAACATATACCGCTAAG3'
KL111_wzy_typing_rev	5'CATTGGACTCTCTATAATTCACTG3'
